# Synthesis of a New α-Azidomethyl Styrene from Safrole via a Dearomative Rearrangement

**DOI:** 10.3390/m1713

**Published:** 2023-08-16

**Authors:** Stephen R. Isbel, Alejandro Bugarin

**Affiliations:** Department of Chemistry and Physics, Florida Gulf Coast University, 10501 FGCU Boulevard South, Fort Myers, FL 33965, USA

**Keywords:** safrole, alkene, allylic azide, styrene

## Abstract

There is a growing interest in developing more efficient synthetic alternatives for the synthesis of nitrogen-containing allylic compounds. This article presents a straightforward two-step protocol to produce 5-(3-azidoprop-1-en-2-yl)benzo[*d*][1,3]dioxole **4** from the natural product safrole. The method yielded the expected α-azidomethyl styrene **4**, in good yield, via a dearomative rearrangement.

## 1. Introduction

Nitrogen is commonly found in natural products and biologically active compounds, making nitrogen-containing compounds essential scaffolds for the development of pharmaceuticals and other biologically relevant molecules [1–3]. In addition, nitrogen-containing allylic compounds play a crucial role in various syntheses, especially in medicinal chemistry [4–6]. There is ongoing research aimed at developing a versatile synthetic method for the production of organic azides [7–10]. Allylic azides are highly valuable starting materials, intermediates, and building blocks in a wide range of organic transformations [11–13]. Traditionally, the synthesis of allylic azides involves alkyl halides’ substitution with sodium azide, albeit prior synthesis of those alkyl halides is required [2], [4–6,10,14,15]. Alternatively, chemists have explored the use of allylic alcohols as starting materials, employing transition metal catalysts (such as Cu [16], Ag [17], and Pd [18,19]), Lewis acids, [20] or triphenylphosphine [21] to activate allylic alcohols. Another method reported by Toste via the hydroazidation of allenes to produce allylic azides utilizes expensive gold as a catalyst [22]. A few years back, Topczewski reported the direct conversion of aldehydes and ketones into allylic azides [23]. Furthermore, the synthesis of allylic azide from alkenes has been accomplished through C–H oxidation. One example is the direct azidation of certain alkenes, as reported by Jiang [24]. Jiang’s method utilizes palladium catalysts in conjunction with molecular oxygen and affords moderate yields after a 24 h reaction period. This study presents a simplified approach to synthesize 5-(3-azidoprop-1-en-2-yl)benzo[*d*][1,3]dioxole **4** from the versatile natural product safrole.

## 2. Results and Discussion

**Scheme 1.**
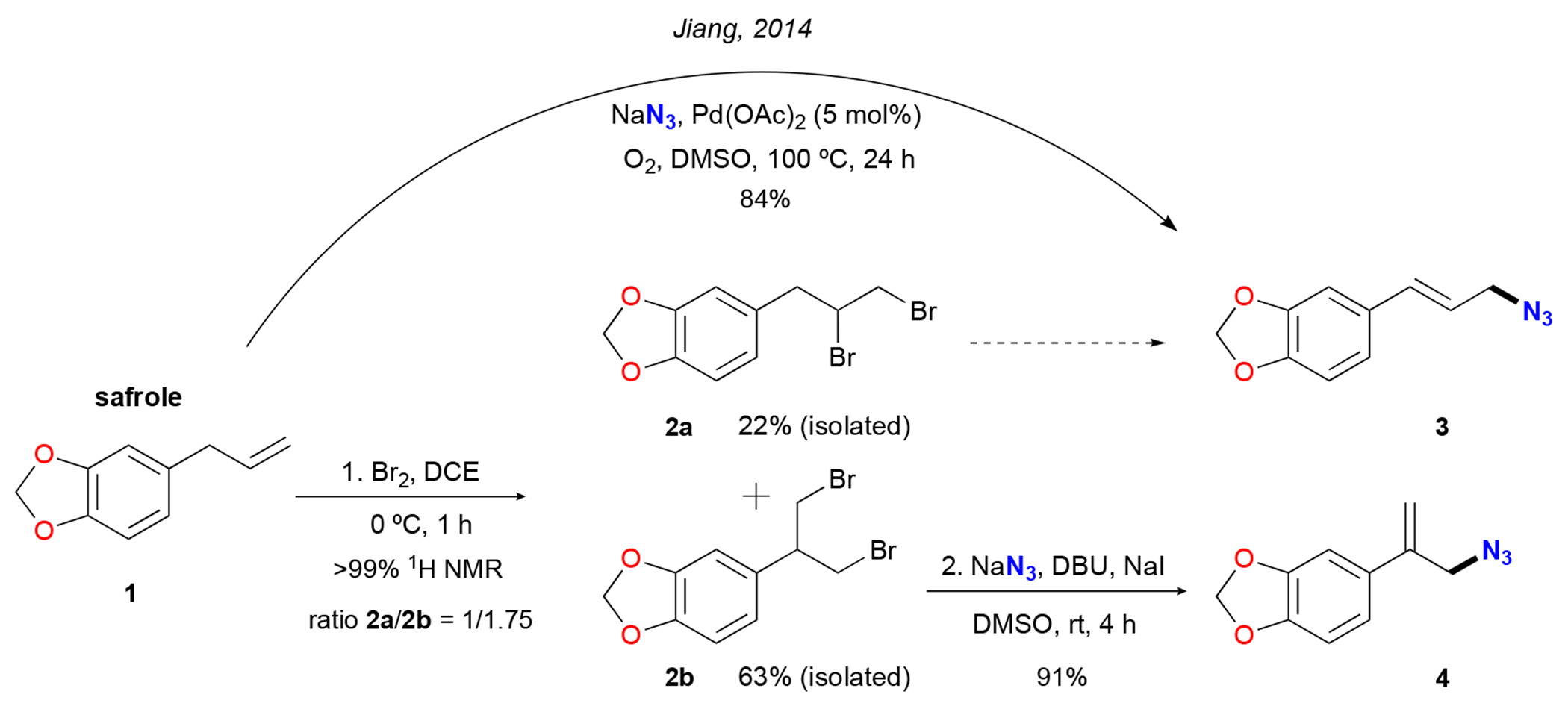
Two-step synthesis of 5-(3-azidoprop-1-en-2-yl)benzo[*d*][1,3]dioxole **4** and Jiang’s direct synthesis of **3** [24].

In addition to the selected synthetic approaches identified above, we have also devoted time and resources to the development of a transition-metal-free method for the straightforward production of allylic compounds from terminal alkenes [25]. Our research efforts have led to a better understanding of the electronic effects in this type of transformation. Initially, it was found that allylaryl bearing electron poor and electron-neutral functional groups favors the formation of (*E*)-allylic compounds such as (*E*)-allylic azide **3** (Scheme 1) [26]. On the other hand, in electron-rich systems (e.g., estragole or safrole), a dearomative rearrangement occurs to preferentially form adducts such as **4** (α-azidomethyl styrene) [27]. At the same time, it was also observed that the formation of regioisomers mainly occurred during the bromination step [28]. Therefore, we conducted a small study to validate that statement. Herein, it was found that, using 1,2-dichloroethane (DCE) as the solvent during the bromination step, the reaction was deemed complete (by ^1^H NMR) after only 20 min at room temperature [29], albeit the **2a/2b** ratio was 1:1.2. However, when the reaction was run at 0 °C, the **2a/2b** ratio changed to 1:1.75, in favor of the rearrangement adduct **2b**, but the reaction time increased to 1 h. Dichloromethane and chloroform were also effective solvents providing quantitative yields after 1 h. The ratios of the products **2a** and **2b** using those solvents were 1:1.43 and 1:1.38, respectively (Scheme 1). Hence, the bromination of safrole **1** at 0 °C using DCE was isolated via column chromatography to produce pure adducts. 5-(2,3-dibromopropyl)benzo[*d*][1,3]dioxole (**2a**) was obtained in a 22% yield, while 5-(1,3-dibromopropan-2-yl)benzo[*d*][1,3]dioxole (**2b**) was isolated in a 63% yield. 1,3-dibromide **2b** was then reacted under our standard azidation conditions [26,27] using sodium azide as the nucleophile and 1,8-diazabicyclo [5.4.0]undec-7-ene (DBU) as the base. To our delight, the expected product [5-(3-azidoprop-1-en-2-yl)benzo[*d*][1,3]dioxole **4**] was obtained in a 91% yield (Scheme 1). The spectra of compound **4** can be found in the Supplementary Materials. In addition, it is worth noting that full mechanism details can be found in our prior reports [25–27].

This study serves as a valuable addition to Jiang’s research, in which safrole was employed to synthesize (*E*)-5-(3-azidoprop-1-en-1-yl)benzo[*d*][1,3]dioxole **3** [24]. In our work, we were able to produce the other regioisomer, namely 5-(3-azidoprop-1-en-2-yl)benzo[*d*][1,3]dioxole **4**, without the requirement of a transition metal catalyst. If needed, accessing allylic azide **3** [30] using our protocol [26] is also possible (Scheme 1). This highlights the versatility and significance of our method in accessing different regioisomers of the desired product.

## 3. Materials and Methods

### 3.1. General Information

All reactions were carried out under air in oven-dried glassware with magnetic stirring at room temperature. Safrole was purchased from Millipore Sigma (St. Louis, MO, USA) and used as received. All reagents and solvents were purchased from Fisher (Hampton, NH, USA) and used as received. Purification of reaction products was carried out via flash column chromatography using silica gel 60 (230–400 mesh). TLC visualization was accompanied by UV light. Concentration in vacuo refers to the removal of volatile solvent using a rotary evaporator attached to a dry diaphragm pump (10–15 mm Hg) followed by pumping to a constant weight with an oil pump (<300 mTorr).

^1^H NMR spectra were recorded at 400 MHZ (Jeol, Akishima, Tokyio, Japan), and are reported relative to CDCl_3_ (δ = 7.26). ^1^H NMR coupling constants (*J*) are reported in Hertz (Hz) and multiplicities are indicated as follows: s (singlet), d (doublet), t (triplet), m (multiplet). Proton-decoupled ^13^C NMR spectra were recorded at 100 MHz and are reported relative to CDCl_3_ (δ = 77). IR experiments were recorded with neat samples on a Jasco FT/IR-4700 (Easton, MD, USA) fitted with diamond ATR sample plate. GCMS data were recorded on a Shimadzu GC-2010 plus (Kyoto, Kyoto, Japan) system (GCMS-QP2010 SE). Elemental Analysis measurement was recorded on a FlashSmart elemental analyzer (Thermo Fisher Scientific, Waltham, MA, USA).

### 3.2. Synthesis of Dibromide Derivatives **2a** and **2b**

Compounds **2a** and **2b** were synthesized via a modification of procedures described in the literature [25,29]: To a 100 mL round-bottomed flask, equipped with a stir bar, was added safrole (3.3 mmol, 1.0 equiv., 0.49 mL) and DCE (17 mL) at room temperature under air. The mixture was cooled down to 0 °C (ice bath), followed by dropwise addition of Br_2_ (3.6 mmol, 0.18 mL, 1.1 equiv.) in DCE (1.7 mL). The reaction was stirred at 0 °C for 60 min. Then, the mixture was concentrated in vacuo. Purification using flash chromatography [silica gel, Hexanes/Chloroform (98:2)] provided both dibromo products (**2a** and **2b**) as separable mixture of regioisomers with 1:1.75 ratio (**2a**:**2b**)

#### *Minor* [colorless oil (220 mg, 22%)]: 5-(2,3-dibromopropyl)benzo[*d*][1,3]dioxole (**2a**):

^1^H NMR (400 MHz, CDCl_3_) 6.79–6.69 (m, 3H), 5.98 (s, 2H), 4.45 (m, 1H), 3.85 (dd, *J* = 11.1, 5.3 Hz, 1H), 3.72 (dd, *J* = 8.5, 2.0 Hz, 1H), 3.60 (dd, *J* = 10.5, 4.5 Hz, 1H), 3.00 (dd, *J* = 13.7, 5.0 Hz, 1H). ^13^C NMR (101 MHz, CDCl_3_) δ 147.7, 147.22, 129.78, 114.83, 112.85, 111.47, 101.84, 51.13, 43.01, 36.93. IR (neat, cm^−1^): 2896, 1619, 1473, 1407, 1230, 1033, 964, 852. LRMS (EI) Calcd for C_10_H_10_Br_2_O_2_ [M], 321.90. Found: 320[M − 2], 322[M], 324[M + 2]. The analytic data are in accordance with those reported in the literature [31].

#### *Major* [colorless oil (630 mg, 63%)]: 5-(1,3-dibromopropan-2-yl)benzo[*d*][1,3]dioxole (**2b**):

^1^H NMR (400 MHz, CDCl_3_) δ 6.79 (d, *J* = 7.6 Hz, 2H), 6.72–6.67 (m, 2H), 5.97 (s, 2H), 3.75 (dd, *J* = 13.6, 6.9 Hz, 2H), 3.66 (dd, *J* = 10.3, 6.4 Hz, 2H), 3.29 (pent, *J* = 13.2, 6.6 Hz, 1H). ^13^C NMR (101 MHz, CDCl_3_) δ 147.81, 147.13, 133.20, 120.96, 108.4, 107.65, 101.18, 48.69, 35.59. IR (neat, cm^−1^): 2888, 1608, 1488, 1438, 1238, 1033, 929, 806. LRMS (EI) Calcd for C10H10Br2O2 [M], 321.90. Found: 320[M − 2], 322[M], 324[M + 2]. The spectroscopic data are in accordance with those reported in the literature [29].

### 3.3. Synthesis of Organic Azide **4**

The new compound 5-(3-azidoprop-1-en-2-yl)benzo[*d*][1,3]dioxole **4** was synthesized via a modification of a procedure described in the literature [27]: To a 10 mL round-bottomed flask, equipped with a stir bar, the pure 1,3-dibromo adduct **2b** (0.50 mmol, 1.0 equiv., 161 mg), DMSO (1 mL), sodium azide (0.60 mmol, 1.2 equiv., 39 mg), NaI (0.50 mmol, 1.0 equiv., 75 mg), and DBU (0.55 mmol, 1.1 equiv., 82 μL) were added sequentially. The reaction flask was capped and stirred at rt for 4 h. Purification via flash chromatography [silica gel, Hexanes/EtOAc (90:10)] provided the pure product **4** as colorless oil (92 mg, 91% yield).

^1^H NMR (400 MHz, CDCl_3_) δ 6.96–6.92 (m, 2H, ArH), 6.80 (d, *J* = 8.0 Hz, 1H, ArH), 5.98 (s, 2H, OCH_2_O), 5.51 (s, 1H, HC=C), 5.26 (s, 1H, HC=C), 4.12 (s, 2H, CCH_2_N_3_). ^13^C NMR (101 MHz, CDCl_3_) δ 147.92, 147.65, 141.16, 132.13, 119.67, 115.25, 108.23, 106.46, 101.21, 55.14. IR (neat, cm^−1^): 3085, 2892, 2094, 1068, 1492, 1230, 1037, 813. LRMS (EI) Calcd for C_10_H_9_N_3_O_2_ [M], 203.07. Found: 203 [M]. Elemental analysis calculated (%) for C_10_H_9_N_3_O_2_: C 59.11, H 4.46, N 20.68. Found: C 59.09, H 4.44, N 20.63.

## 4. Conclusions

In summary, we have introduced a milder two-step approach for the synthesis of 5-(3-azidoprop-1-en-2-yl)benzo[*d*][1,3]dioxole **4** from safrole. The method is straightforward. The obtained yield in our study compares favorably with that of existing strategies based on allylic C-H activation. Notably, our protocol exclusively yields the expected styrene product. This methodology offers a milder, cost-effective, selective, and simpler alternative for efficiently constructing styrene azide scaffolds from electron-rich systems.

## Supplementary Material

SI

## Data Availability

The data are contained within the article.
