# Supplementary material for: Synthesis of a New α-Azidomethyl Styrene from Safrole via a Dearomative Rearrangement
Source: Molbank. Author manuscript; Available in PMC 2023 Nov 2. (PMC10621569; doi:10.3390/m1713)
Supplement: SI [file NIHMS1939887-supplement-SI.docx]

**Synthesis of a New α-Azidomethyl Styrene from Safrole via Dearomative Rearrangement**

Stephen R. Isbel and Alejandro Bugarin*

* Department of Chemistry and Physics, Florida Gulf Coast University,

10501 FGCU Boulevard South, Fort Myers, FL 33965

[abugarin@fgcu.edu](mailto:abugarin@fgcu.edu)

**Table of Contents**

^1^H and ^13^C NMR Spectra of Compound **4** ………….……….………………… S-2

Mass Spectrum of Compound **4** …………………….……….………………… S-3

IR Spectrum of Compound **4** ………….……………….…….………………… S-4

Elemental Analysis Chromatogram of Compound **4**………….…………. …… S-5

**Figure S1.** ^1^H NMR (400 MHz, CDCl_3_)

CDCl_3_

H_2_O

**4**

**Figure S2.** ^13^C NMR (101 MHz, CDCl_3_)

**4**

**Figure S3.** Mass Spectrum of Compound **4**


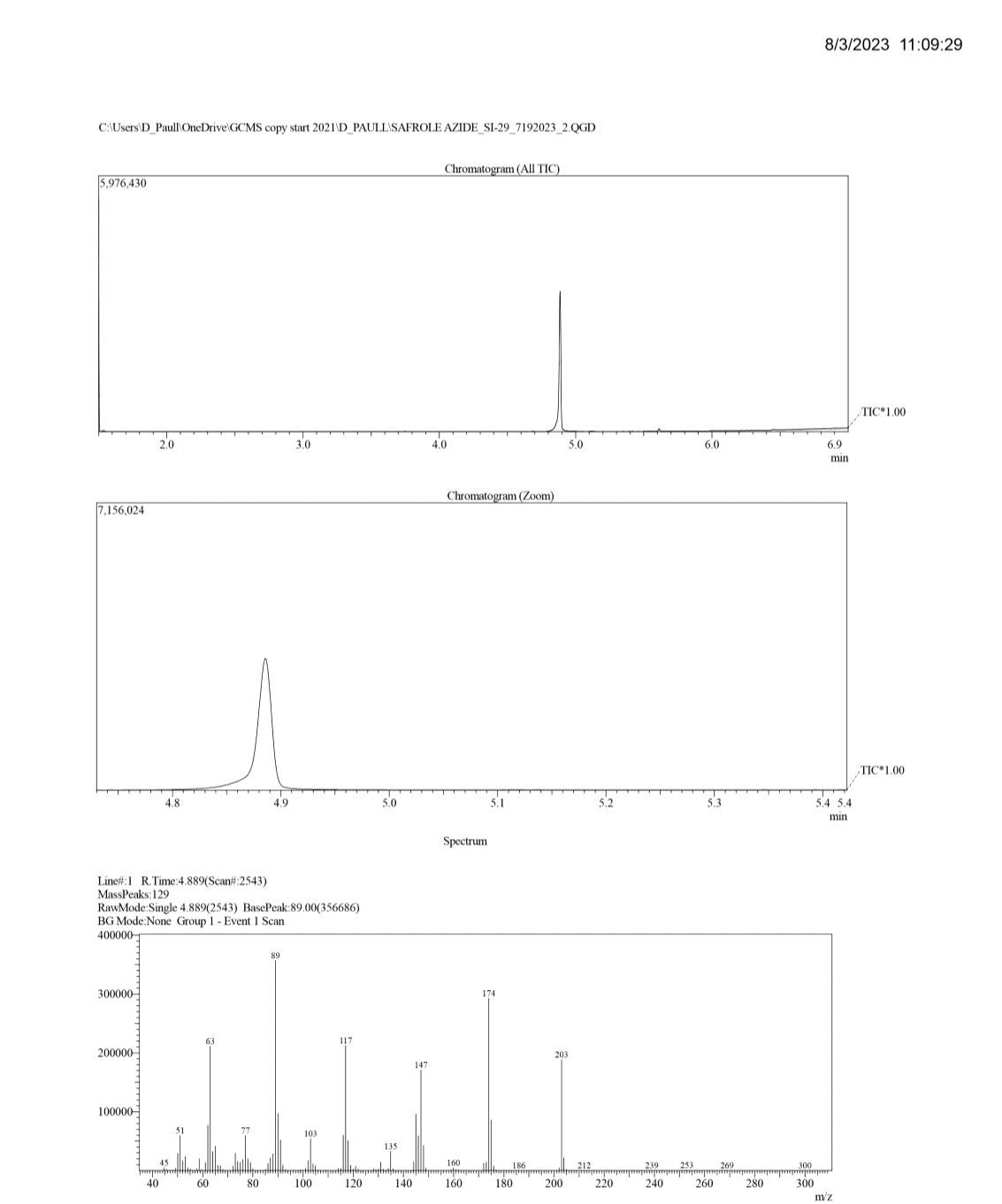


**Figure S4.** IR Spectrum of Compound **4**

**Figure S5.** Elemental Analysis Chromatogram of Compound **4**

|  |  | Nitrogen | Carbon | Hydrogen | Sulphur |
| --- | --- | --- | --- | --- | --- |
| 1 | bypass | 0 | 0 | 0 | 0 |
| 2 | blank | 0 | 0 | 0 | 0 |
| 3 | Std: BBOT | 6.51 | 72.53 | 6.09 | 0 |
| 5 | Compound **4** | 20.63 | 59.09 | 4.44 |  |
